# Diagnostic Evaluation Using Salivary Gland Ultrasonography in Primary Sjögren’s Syndrome

**DOI:** 10.3390/jcm12062428

**Published:** 2023-03-22

**Authors:** Yen-Fu Chen, Ao-Ho Hsieh, Yao-Fan Fang, Chang-Fu Kuo

**Affiliations:** 1 Division of Rheumatology, Allergy and Immunology, Department of Internal Medicine, Chang Gung Memorial Hospital, Taoyuan 333, Taiwan; 2 Center for Artificial Intelligence in Medicine, Chang Gung Memorial Hospital, Taoyuan 333, Taiwan

**Keywords:** primary Sjögren’s syndrome, salivary gland ultrasonography, IL-1 family cytokines

## Abstract

The purpose of this study is to investigate the clinical manifestations in patients with early primary Sjögren’s syndrome (pSS) based on the severity score found by salivary gland ultrasonography. Thirty-five newly diagnosed patients with early pSS were enrolled and divided into mild (score 0–1) and severe (score 2–3) groups according to the salivary gland ultrasonography grade (SGUS) scores at baseline. Clinical evaluation, ESSPRI and ESSDAI index values, sicca symptoms of the mouth, salivary capacity, and serum autoantibodies and cytokines were investigated. The mean age of pSS patients at diagnosis was 49.9 ± 11.9 years, and the mean duration of sicca symptoms was 0.58 years. ESSPRI (EULAR Sjögren’s syndrome patient report index) and ESSDAI (EULAR Sjögren’s syndrome disease index) scores were 15.97 and 4.77, respectively. Clinical manifestations, including the low production of saliva and autoantibody production, such as antinuclear antibodies, rheumatoid factor, and anti-SSA antibody, were found. A higher prevalence of rheumatoid factor (*p* = 0.0365) and antinuclear antibody (*p* = 0.0063) and a higher elevation of total IgG (*p* = 0.0365) were found in the severe group than in the mild group. In addition, the elevated titer of IL-25 was detected in the severe group than in the mild group. This observation indicated that salivary gland ultrasonography grade (SGUS) scans may help physicians diagnose pSS and the elevated titer of IL-25 in patients may be implicated in the pathogenesis of pSS.

## 1. Introduction

Primary Sjögren’s syndrome (pSS) is a chronic autoimmune disease characterized by lymphocytic infiltration of the exocrine glands, the presence of an antibody to SSA/Ro and/or SSB/La, and hypergammaglobulinemia [1]. Dryness is the main symptom, albeit a proportion of patients experience other symptoms including fatigue, arthralgia, and arthritis, or extra glandular manifestations such as lymphomas [2,3,4]. The occurrence of pSS is rarer in men and predominantly occurs in elderly women [5]. However, the etiology and pathogenesis of pSS remain largely unknown, and there is no effective treatment to cure this disease so far.

Sialo scintigraphy (sSC) and sialography in the American-European classification criteria are the first line and alternative diagnostic imaging tools for clinical SS diagnosis [6]. In recent decades, salivary gland ultrasonography (SGUS), another noninvasive imaging method, has emerged as a useful tool for the evaluation of major salivary gland lesion in pSS and serves as an auxiliary examination to salivary gland biopsy [7,8,9,10]. US has a high specificity and is accurate in establishing a diagnosis of pSS comparable to sSC and biopsy [11,12].

Cytokines act as essential factors in mediating immune cell response and contribute to exocrine tissue damage [13]. The IL-1 family has important roles in activating and reinforcing the function of polarized T cells. The overexpression of IL-18 in patients with SS is involved in the activation of Th1 cells and NK cells [14,15,16]. IL-33 drives naïve T cells toward Th2 cells through activation of dendritic cells (DCs), and IL-1 plays a key role in Th17 cell differentiation and maintenance [17]. This correlation between IL-18 and anti-SSA/SSB autoantibodies has been described and is considered as a risk factor for the development of lymphoma [18,19]. Several studies have demonstrated the role of the IL-33/soluble ST2 (sST2) level in patients with pSS [20,21]. In addition, Th17 cytokines, IL-17A and IL-17E (IL-25) may be involved in the pathogenesis of pSS [22]. An increased level of IL-17 in the serum, saliva, tears, and glandular tissue of patients with pSS was found [23,24]. The presence of elevated IL-17 is correlated with total IgG level, autoantibody production, and lymphocytic infiltrates, and disease activity in pSS has been reported [16,25,26]. Moreover, a high level of IL-25 mRNA expression identified in the labial salivary gland (LSG) is relevant to lymphocytic infiltration [27].

In the current study, we diagnosed patients with early pSS using a simplified scoring system and investigated the clinical manifestation and serum cytokine release, such as IL-17, IL-18, IL-25, IL-31, and IL-33, in patients. We found that the ultrasonography parameters for the evaluation of salivary gland disease are correlated with the clinical manifestations and serum cytokines in patients with pSS.

## 2. Material and Methods

### 2.1. Patients

All participants were enrolled from rheumatology clinics in the Division of Rheumatology, Allergy, and Immunology in Linkou Chang Gung Memorial Hospital. The diagnosis of patients with Sjögren’s syndrome was based on the 2002 AECG criteria for the classification and diagnosis of pSS (Schirmer test <5 mm and positive anti-SSA). This study was approved by the Institutional Review Board of Chang Gung Memorial Hospital, and all the participants, comprising 35 patients with pSS (mean ± SD, 49.9 ± 11.9) and 32 healthy controls (mean ± SD, 48.6 ± 10.2), provided written informed consent before their inclusion as required by the Declaration of Helsinki (approval numbers # 201601658B0D001 and 201600795B0D001). The follow-up sera and clinical information were collected from rheumatic patients whose disease was well controlled after disease onset.

### 2.2. Cytokine Detection

The measurements of serum IL-17A, IL-18, IL-25, IL-31, and IL-33 were performed according to the manufacturer’s instructions (Abnova, Taipei City, Taiwan; Catalog code: KA-3073, KA-0561, KA-2190, KA-3863, and KA-3410). The calibration range is 31.2–2000 pg/mL (IL-17A, IL-18, and IL-25), 31.2–1000 pg/mL (IL-31), and 15.6–1000 pg/mL (IL-33). The inter- and intra-assay CVs are 5.2% and 2.5%, respectively.

### 2.3. Ultrasonography for the Major Salivary Glands

**Figure 1:**
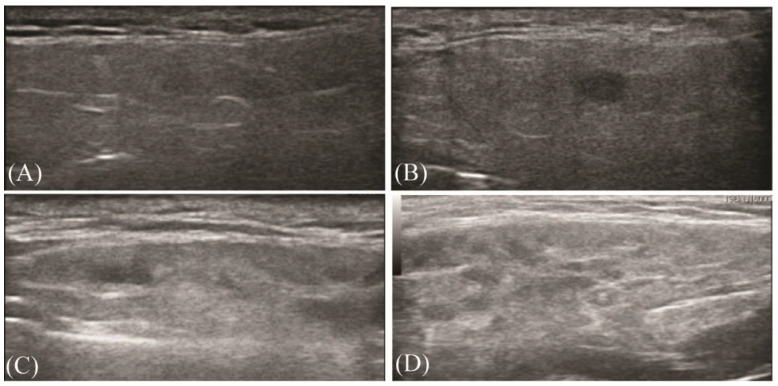
Ultrasonographic images of four parotid glands illustrating varying grades of nonspecific to pathological changes. Scores of 0–3 were determined by evaluation of US examination of each patient’s parotid glands. (**A**) Normal homogenous parotid gland (score 0), (**B**) mild inhomogeneous parotid gland (score 1), (**C**) evident inhomogeneous parotid gland (score 2), and (**D**) grossly inhomogeneous parotid gland (score 3). Scores of 0–1 were considered as normal or nonspecific changes, and scores of 2–3 were diagnosed as pathological changes, which were related to primary SS.

The parotid and submandibular glands were examined through US using a ACUSON P300 system with a linear high-frequency transducer (LA435, 6–18 MHz). The parotid glands and the submandibular glands were evaluated in a cross-sectional plane. The US examination was performed by one out of two rheumatologists. Representative US images were stored digitally as jpg images on a standard high-quality computer screen. Images were evaluated by two rheumatologists who were both blinded for clinical diagnosis or laboratory examination, including subjective sicca symptoms, salivation function test, evaluation of ESSDAI and ESSPRI, serum autoantibodies and cytokines. The glandular homogeneity and presence of hypoechogenic areas were evaluated according to the scoring system (score 0–3) from Hocevar’s study [9,10]. A score 0–1 represents normal/non-specific changes, and a score of 2–3 represents pathological changes (Figure 1).

### 2.4. Clinical Evaluation

Patient specimens were collected on the patient’s first visit to our hospital. All investigations were conducted at a single point in time, including clinical measurements and cytokine measurement. The measurements of serum autoantibody titer, rheumatoid factor (RF), complement component 3/4 (C3/C4), and salvia production were performed by the Department of Laboratory Medicine in Chang Gung Memorial Hospital. Briefly, the measurement of serum RF IgM was performed using an ELISA specific test. A specimen unit value ≤ 6 is considered as an RF-negative response. The autoantibodies for SSA/SSB, Sm. RNP, and dsDNA were evaluated by the ANA-11 plus test system (AtheNA multi-Lyte, Product Number: A21101). Specimen unit values for each of the multiplexed analytes of greater than 120 are considered positive specimens. The examination of the antinuclear antibody was conducted by ANA-HEp2 (AESKUSLIDES, Diagnostics). The screen titer is recommended at a dilution of 1:80 for adult samples.

### 2.5. Scores of ESSDAI and ESSPRI

The score for assessment of disease activity was determined using ESSPRI and ESSDAI. ESSPRI is a patient self-reported outcome completed by patients using a visual scale (Patient’s Global Assessment–PGA) before their evaluation. ESSDAI is a physician-administered disease activity index used to evaluate systemic complications during their visit.

### 2.6. Subjective Sicca Symptoms and Fatigue

The severity of sicca symptoms of the mouth and eyes was reported by patients on a visual analogue scale (VAS) ranging from 0 to 10, and the level of fatigue was reported on a VAS ranging from 0 to 100, with a higher number representing more severe symptoms. Recordings of fatigue were measured using a graded ruler and recordings of sicca symptoms were given on a graded scale from 0 to 10.

### 2.7. Saliva Production

Salivary gland functional capacity was evaluated with saliva production (gram/2 min) by a clinical laboratory technologist, and the tests were performed with the patients fasting for 3.5 h before examination. Saliva was obtained by letting patients chew on 2 × 2-inch sterile gauze for 15 min [28]. The gauze with saliva was collected and the volume of secreted saliva was determined by weighing, with 1 g of saliva corresponding to 1 mL. Levels of ≤1.5 mL/15 min of saliva production were considered pathologically reduced.

### 2.8. Statistical Analysis

Statistical analyses of the titers and multiple-comparison corrections were performed using the Prism software 8.0 (GraphPad Software, Boston, MA, USA). Student’s *t* and two-tailed Fisher’s tests were used for these comparisons, with graphs depicting the mean ± SEM. The independent *t*-test, Kruskal–Wallis test, and chi-squared test were used to examine the results of the serological analysis. A *p*-value < 0.05 was considered significant, and different levels of significance were reported (* *p* ≤ 0.05; ** *p* ≤ 0.01; *** *p* ≤ 0.001).

## 3. Results

### 3.1. Ultrasonographic Evaluation of Major Salivary Glands

Clinical examination was performed in thirty-five patients with pSS (Table 1). The presence of hypoechogenic areas and glandular homogeneity in salivary glands were evaluated and scored as 0–1 (normal or non-pathogenic stage) or 2–3 (pathogenic change). The severe group showed a greater hypoechoic area and a different degree of glandular inhomogeneity in both the submandibular (*p* < 0.0001) and parotid glands (*p* < 0.0001) than the mild group (Table 1). Only one patient in the severe group (1/15, 6.67%) with normal submandibular gland grade (score 0–1) showed pathogenic change in the parotid gland (score 2–3). There were no significant differences shown in age of onset, gender, and symptoms duration.

### 3.2. ESSDAI and ESSPRI Index Values

To investigate the correlation of gland affection and disease activity, the ESSDAI and ESSPRI scores were calculated (Table 1). In the ESSPRI, our results showed that sicca syndromes, fatigue, and arthralgia were urgent to be improved in 61%, 57%, and 43% of patients with Sjögren syndrome, respectively, but the ESSPRI and ESSDAI scores showed no significant differences between the severe and mild groups. All patients were non-smoking, non-drinking, and no other comorbidities were mentioned.

### 3.3. Serological Analysis

We further investigated the association between autoantibody production and gland affection. In total, 88.6% of patients (31/35) with pSS were anti-SSA-antibody-positive. A total of 68.6% of patients (24/35) were ANA-seropositive, and 40.0% of anti-SSB-antibody-positive patients (14/35) were also ANA-positive (Table 1). Of all the patients, 40.0% and 40.0% were serum rheumatoid factor (RF)-positive (14/35), with an increasing titer of total IgG (14/35). A low proportion of patients presented with a low level of C3 or C4, dsDNA-positivity, or an elevated titer of IgG_4_. To compare the clinical manifestation between the mild and severe groups, a higher prevalence of serum RF (*p* = 0.0365) or ANA (*p* = 0.0063) was observed in the severe group than in the mild group. Moreover, a low level of saliva production (*p* = 0.0050) was observed in the severe group.

### 3.4. Cytokine Detection

**Figure 2:**
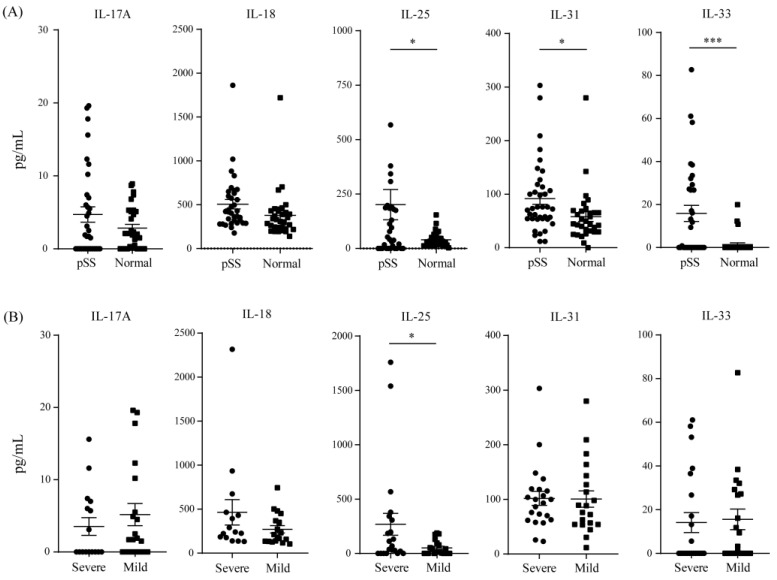
The measurement of serum level of IL-17, IL-18, IL-25, IL-31, and IL-33 by using sera from patients with pSS and healthy controls. (**A**) pSS patients vs. healthy controls. (**B**) Severe group vs. mild group. A *p*-value < 0.05 was considered significant, and different levels of significance were reported (* *p* ≤ 0.05; *** *p* ≤ 0.001).

The measurement of IL-17, IL-18, IL-25, IL-31, and IL-33 were performed using sera from patients with pSS (n = 35) and healthy controls (n = 32) (Figure 2). The elevated titers of IL-25 (*p* = 0.0256), IL-31 (*p* = 0.0227), and IL-33 (*p* < 0.0005) were found in patients with pSS compared to healthy controls. The level of IL-17 and IL-25 showed no significant differences between the two groups. In the comparison of the severe (n = 15) and mild groups (n = 20), we found that the levels of cytokines showed no significant differences, except for IL-25 (*p* = 0.0492).

## 4. Discussion

Primary SS diagnosis is based on a combination of clinical, serologic, histologic, functional, and instrumental parameters to evaluate the autoimmune exocrinopathy in the salivary and lachrymal glands and the systemic autoimmune response [7,8]. Sialography and scintigraphy in salivary gland diseases are currently clinical methods to assess the involvement of major salivary glands [9,10]. For now, salivary gland ultrasonography (SGUS) is considered an easy and noninvasive image technique to assess the degree of salivary gland involvement in pSS [11,12]. De Vita et al. reported that glandular inhomogeneity is a critical discriminating characteristic to distinguish between patients with SS and healthy individuals [29]. The appearance of gross parenchymal inhomogeneity with scattered hypoechoic areas caused by lymphocytic infiltration is specific for the diagnosis of pSS. For this purpose, several discriminating elements, including gland size, border visibility, parenchymal echogenicity and inhomogeneity size, glandular calcifications, and hyperechogenic bands, were optionally brought into current scoring systems [30,31]. In the current study, we examined the submandibular salivary gland by SGUS using a simplified scoring method for the assessment of glandular homogeneity and hypoechogenic areas. Forty-six patients were first diagnosed with pSS and separated into severe and mild groups based upon the SGUS scoring. Despite some patients appearing to develop lower titers of anti-SSA antibodies and unobvious clinical manifestation on the first visit, they were all diagnosed with pSS in the six months. In addition, we found that severe group patients have a higher ESSDAI score, a significant prevalence of rheumatoid factor and antinuclear antibody, and a lower production of saliva, compared with mild patients. This observation suggests that SGUS may help physicians to distinguish patients with pSS from individuals with dryness in the initial stage of disease onset.

In the clinical evaluation, 88.6% of pSS patients were anti-SSA-positive, and a higher prevalence of RF and anti-SSB positivity were observed in the severe group compared to the mild group. A lower ANA-positive rate was found in the mild group than in the severe group, suggesting that this observation may be due to the cases of suspected Sjögren’s syndrome or the bias due to the difference in methodology. All patients were negative for CRP and antibodies for RNP and Sm. Of our patients, one patient, a 65 y/o woman, developed MALT lymphoma. According to the patient’s statement, the right parotid gland underwent enlargement after around half a year, and dry eyes and a dry mouth were also mentioned.

Cytokines play a central role in the initiation and perpetuation of pSS. Altered cytokines, such as IL-1α/β, IL-6, IL-8, IL-17A, IL-23, TNF-a, and IFN-γ, in the serum or saliva of patients with pSS have been extensively discussed [25,32]. However, the expression of serum IL-25, IL-31, and IL-33 in the primary SS has been less reported previously. IL-31 and IL-33 are new cytokines belonging to the IL-1 family including IL-18 and IL-1α/β. The increased level of IL-18 in circulation and the saliva gland was suggested to modulate the immune inflammatory pathways contributing to the pathogenesis of pSS [16,18]. Additionally, the level of IL-18 was reported to positively correlate with the titer of anti-SSA/SSB antibody and rheumatoid factor in primary SS patients [33]. The elevated serum level of IL-33 and/or its decoy receptor, soluble ST2 (sST2), was observed in sera and saliva gland tissue in the pSS patients [20,34]. IL-31 is a pro-inflammatory cytokine, primarily expressed in Th2 cells, that promotes the expression of IL-6 and IL-8 and takes part in tissue remodeling but is less addressed in pSS [35]. Herein, we examined the serum titer of IL-17A, IL-18, IL-25, IL-31, and IL-33 from primary SS patients before medical treatment and healthy individuals. The recruiting of healthy individuals was considered as a baseline of cytokine expression in the normal statues. We observed significant elevation of IL-25, IL-31, and IL-33 in patients with pSS compared with normal individuals. This observation suggests that IL-25, IL-31, and IL-33 may play a role in the early stage of pSS.

The increased titer of IL-17A has been reported to modulate the primary SS [25,36]. IL-17A level is correlated with the severity of lymphocytic infiltration in the labial glands of SS patients [37]. The increased level of IL-17A was also observed in the serum, tears, or saliva from SS patients, suggesting that the involvement of inflammatory cytokines may contribute to clinical manifestation in patients [25,28,38]. However, some studies have shown contradictory results regarding the serum level of IL-17A in primary SS patients [22,37]. Nguyan et al. reported that the immunohistochemical stains for salivary gland biopsy from SS patients exhibited strong positive staining for IL-17A within lymphocytic foci and diffuse staining on epithelial cells, but this observation contrasted with results of the IL-17A level in serum and saliva from SS patients [24]. In animals, IL-17A is highly expressed in the glandular tissue in association with lymphocytic foci of submandibular glands, according to a mouse model for pSS but is not detectable in the saliva or serum in early stages of animal experiments. In addition, it has been reported that serum IL-17A was only increased in patients with longer disease duration and presented less correlation with clinical features in pSS patients [22,39]. In the current study, these patients were newly diagnosed with primary SS without systemic corticosteroids or immunosuppressive agent treatment. The lower disease duration and mean ESSDAI were mentioned. This may be one possible reason that IL-17A serum level was not shown to be significantly increased compared to the control subjects. On the other hand, we compared the expression levels of serum cytokines between the severe and mild groups but found no significant differences between the two groups. This result implied that SGUS is a useful tool to diagnose the histopathological changes in the saliva gland in the early stages of pSS from healthy individuals. However, a combination of clinical, serological, and instrument parameters may provide sufficient information for attending physicians to evaluate the disease progression of pSS.

Several studies have tried to stratify pSS patients and link the disease manifestation based upon clinical parameters, for example, positivity of antibodies, levels of total IgG/IgG subclass, or pathogenic change in salivary glands to gain better knowledge of this disease [40,41,42]. Relationships between IL-18 in the serum or salivary gland, autoantibody production, and periductal inflammatory cell infiltration have been reported [18]. Serum IL-18 level is significantly higher in anti-SSA/SSB-positive primary SS patients than in anti-SSA/SSB-negative patients and showed positive correlation with anti-SSA and anti-SSB titers. Moreover, immunohistochemical stains revealed that IL-18 was expressed by CD68^+^ macrophages in germinal-center-like structures in SS salivary glands and in normal lymph nodes [18]. In addition, IL-33 is described as an inducer for type 2 immune response to activate helper T cells or innate immune cells such as mast cells [17]. Recent research has revealed that IL-33 can drive the autoantibody generation by the induction of the B cell survival factor (BAFF) from neutrophil and dendritic cells [43]. Notably, chronic exposure to IL-33 may induce expression level of BAFF and promote the elevated numbers of B and T follicular helper cells and the formation of a germinal center. This research may provide a potential association between elevated levels of IL-18 and IL-33 and autoantibody production in pSS patients. In the current study, we observed the elevated titer of IL-25, IL-31, and IL-33 but not IL-18. The correlation between cytokine level and autoantibody titer was not found in the early stage of disease onset.

There were some limitations in our current study. We recruited patients based on the 2002 AECG criteria for the classification and were compatible with 2016 ACR/EULAR classification criteria of Sjögren’s syndrome, especially a positive anti-SSA response and a positive Schirmer test. In addition, we excluded some criteria, for example, smoking, alcohol drinking, or medication use such as antihistamine and diuretics. However, some information about comorbidity was missed here, such as hypertension, diabetes mellitus, etc. Moreover, the healthy controls recruited in this study were considered as the cytokine expression in the normal situation, which was compared to those in pSS patients, but the clinical measurement of the healthy controls was not carried out and discussed in this study.

In the current study, we evaluated the glandular homogeneity and presence of hypoechogenic areas according to Hocevar et al. and later applied in clinical use, according to Theander et al. [10,44]. Recently, the JOUSSE-JS group developed a novel semiquantitative US score in patients with pSS. The intra-reader reliability to detect and score these lesions is considered excellent and inter-reader reliability is good [45]. This may provide a better insight or more choice to investigate and diagnose the progression of Sjögren’s syndrome.

## 5. Conclusions

In the present study, we investigated the clinical manifestations of patients by using salivary gland ultrasonography (SGUS) to diagnose early pSS. Patients were separated into severe and mild groups according to a simplified scoring system. The severe patients had a higher incidence of autoantibody positivity, including rheumatoid factor and antinuclear antibody, than the mild patients did. The elevated titers of IL-25, IL-31, and IL-33 were observed in patients with primary Sjögren’s syndrome compared to the healthy subjects but showed no significant differences between the severe and mild patients. This observation suggests that SGUS may help physicians to distinguish patients with early pSS during the assessment of sicca symptoms.

## Figures and Tables

**Table 1:** Clinical and salivary gland ultrasound features of patients with primary Sjögren’s syndrome.

Characteristics	SS Patients (n = 35)	Mild (n = 20)	Severe (n = 15)	*p*-Value
Mean Age, yr (SD)	49.9 (11.9)	48.5(12.9)	49.5 (10.6)	0.8128 ^a^
Female, n (%)	34 (97.8)	19 (95.0)	15 (100)	1.0000 ^b^
Disease duration, yr (SD)	0.58 (0.32)	0.61 (0.32)	0.53 (0.31)	0.4982 ^a^
ESSDAI, mean (SD)	4.77 (4.82)	3.80 (3.00)	6.07 (6.40)	0.2199 ^a^
Salivary gland ultrasonography scores (SD)	1.17 (0.82)	0.55 (0.51)	2.00 (0.00)	<0.0001 ^a^
Parotid gland, scoring (SD)	1.14 (0.81)	0.55 (0.51)	1.93 (0.26)	<0.0001 ^a^
Submandibular gland, scoring (SD)	1.14 (0.81)	0.55 (0.51)	1.93 (0.95)	<0.0001 ^a^
ESSPRI, mean (SD)	15.97 (6.40)	15.45 (7.20)	16.67 (5.31)	0.5691 ^a^
Sicca syndrome, level score (SD)	6.11 (2.26)	5.85 (2.48)	6.47 (1.96)	0.4169 ^a^
Fatigue, level score (SD)	5.80 (2.70)	5.10 (3.16)	6.73 (1.58)	0.0546 ^a^
Arthralgia, level score (SD)	4.06 (3.63)	4.50 (3.35)	3.25 (3.97)	0.4261 ^a^
RF, n (%)	14 (40.0)	5 (25.0)	9 (60.0)	0.0365 ^c^
Anti-SSA, n (%)	31 (88.6)	18 (90.0)	13 (86.7)	0.7590 ^b^
Anti-SSB, n (%)	14 (40.0)	6 (30.0)	8 (53.3)	0.1632 ^c^
Anti-RNP, n (%)	0 (0)	0 (0)	0 (0)	-
Anti-Sm n (%)	0 (0)	0 (0)	0 (0)	-
Antinuclear, n (%)	24 (68.6)	10 (50.0)	14 (93.3)	0.0063 ^c^
Low C3, n (%)	5 (14.3)	2 (10.0)	3 (20.0)	0.4028 ^c^
Low C4, n (%)	2 (5.7)	0 (0)	2 (13.3)	0.0926 ^b^
Anti-dsDNA, n (%)	1 (2.9)	0 (0)	1 (6.7)	0.2414 ^b^
High IgG, n (%)	14 (40.0)	7 (35.0)	7 (46.7)	0.0365 ^c^
High IgG_4_, n (%)	4 (11.4)	3 (15.0)	1 (6.7)	0.4432 ^c^
ESR, n (%)	7 (20.0)	3 (15.0)	4 (26.7)	0.3932 ^c^
CRP, n (%)	0 (0)	0 (0)	0 (0)	-
Low saliva production, n (%)	10 (28.6)	2 (10.0)	8 (53.3)	0.0050 ^c^

Normal specimen reference ranges. Rheumatoid factor (RF, <15 IU/mL), SSA, SSB, RNP, and Sm (negative < 100 unit, equivocal 100–120); Antinuclear antibody (ANA > 1:80); complement 3 (C3, 80–155 mg/dL); complement 4 (C4, 13–37 mg/dL); dsDNA: (<92.6 unit, equivocal 92.7–138.9); IgG titer(700–1600 mg/dL); IgG4(3–201 mg/dL), erythrocyte sedimentation rate (ESR, female >50 y, 30; female/male <50 y, 20), c-reactive protein (CRP, ≤5 mg/L), and saliva production (>1.5 g, g/15 min). ESSPRI: EULAR Sjögren’s syndrome patient report index), ESSDAI: EULAR Sjögren’s syndrome disease index, RNP: ribonucleoprotein, Sm: Smith, C: complement, dsDNA: double stranded deoxynucleic acid. y: age in years, SD: standard deviation. ^a^ Independent *t*-test ^b^ Fisher’s exact test ^c^ Chi-square test.

## Data Availability

The datasets analyzed during the current study are not available publicly due to our IRB policy but are available from the corresponding author upon reasonable request.
